# Styrene–Butadiene Rubber Latex Modified Mortars Prepared with High Early Strength Portland Cement

**DOI:** 10.3390/ma17236000

**Published:** 2024-12-07

**Authors:** Modestas Kligys, Giedrius Girskas

**Affiliations:** Institute of Building Materials, Faculty of Civil Engineering, Vilnius Gediminas Technical University (VILNIUS TECH), 10223 Vilnius, Lithuania; giedrius.girskas@vilniustech.lt

**Keywords:** cement-based materials, polymer dispersion, modified mortars, styrene–butadiene rubber latex, Portland cement, high early strength class

## Abstract

The increased early hydration rate of high early strength cement has economic advantages in many civil engineering fields (faster formwork removal or earlier demoulding of precast elements). Styrene–butadiene rubber (SBR) latex is the most common polymer in aqueous dispersions suitable for admixing in cement-based materials. It allows the designing of structures with specific properties for a variety of applications. The analysis of literature sources has shown that different properties of SBR latex-modified cement-based material samples reported were usually measured at 3, 7, 14, and 28 days of hardening. In this research, the authors decided to investigate a combined effect of high early strength Portland cement, characterized by an increased hydration rate, and SBR latex able to retard this process for a prolonged hardening period—up to 90 days in modified mortar samples. This study covers the results of the effect of different amounts of SBR latex (5%, 10%, 15%, and 20%) on the properties of modified mortar samples with a constant water-to-cement ratio prepared with high early strength Portland cement 42.5 R. The mortar samples were prepared from local raw materials produced by the Lithuanian companies. The properties, such as dry bulk density, ultrasonic pulse velocity, capillary water absorption, compressive and flexural strengths, and toughness, after three different hardening periods (7, 28, and 90 days) of the mortar samples were investigated. The applied mathematical–statistical methods allowed a detailed prognosis of the dependence between the dry bulk density and the strength properties of modified mortar samples. The combination of 42.5 R strength class Portland cement with the SBR latex in amounts ranging from 5% to 20% seems to be suitable for designing durable structures with specific properties.

## 1. Introduction

The Romans and Greeks discovered the first cement based on the pozzolanic reactions of water, lime, and finely ground volcanic ash [1]. Later, in the 1770s, English civil engineer J. Smeaton discovered a method for producing hydraulic limes, which were used in cement production. The addition of hydraulic limes allowed the formation of stable hydrates that could withstand the influence of the environment [2]. Portland cement was one of the main inventions of the first industrial revolution. This term was first applied by a bricklayer J. Aspdin in the British patent in 1824. The patent described the process for making clinker from lime and clay using the calcination method [3]. The obtained clinker was ground to powder to obtain cement.

Portland cement is the most expensive raw material in the Lithuanian concrete industry. The 2024 prices (calculation per 1 m^3^) are as follows: water (1.00 Eur); chemical admixtures (5.00); sand (5.00 Eur); gravel (16.5 Eur); and Portland cement (30.0 Eur). In the presence of water, Portland cement, due to hydration reactions, binds different types of aggregates into a monolithic structure, resulting in the formation of cement-based materials, such as plasters, mortars, concretes, etc. [1,4,5]. The properties of cement-based materials depend on the properties of hardened cement paste, i.e., on the properties of Portland cement and water-to-cement ratio, on the strength of aggregates, on the strength of the interfacial transition zone, also on the presence of defects and, in particular, on the porosity [1,3]. Cement is classified into three standard strength classes by the minimum 28-day compressive strength: 32.5, 42.5, and 52.5, respectively [6,7]. Each class of standard strength has two classes of early strength, ordinary early strength indicated by the suffix N, and high early strength indicated by the suffix R [8]. The chemical composition of high early strength Portland cement is similar to that of ordinary early strength Portland cement, but the finer grinding of N-class cement (larger specific surface) increases the hydration rate and gives economic advantages in many civil engineering fields (faster formwork removal or earlier demoulding of cast elements).

The properties of mortars, which is a type of cement-based composite, could be improved by adding polymer admixtures, which usually are of four main types: (I) polymer latex (polymer dispersion), (II) dry powder, (III) water-soluble polymer, and (IV) liquid polymer [9]. Liquid dispersions are more effective than dry powders due to the development and uniform distribution of polymer films [10]. Styrene–butadiene rubber (SBR) latex is the most common and widely used polymer in aqueous dispersion. It is admixed to cement in mortars, grouts, and concretes at a typical polymer-to-cement (P:C) ratio, ranging from 0% to 20% [11,12,13]. SBR latex can be added to mortar in two different ways: (I) keeping a constant water-to-cement ratio (W:C) to obtain similar hydration of the cement paste [14,15], and (II) meeting the required viscosity of ordinary mortar, usually by adjusting the W:C ratio [16,17]. The molecular structure of the SBR latex containing the flexible butadiene chains and the rigid styrene chains, the combination of which gives the mortar (at 15% SBR latex content) improved fluidity (by 37–40%), more compact structure, reduced yield stress and viscosity, and also increased porosity [18]. Electromigration tests [11] demonstrate improved resistance of chloride penetration (reduced apparent diffusion coefficients of chloride anion) and reduced general ionic permeability (reduced electric charge) of the SBR latex-modified mortars. Research results [19] have shown that the change in the interfacial transition zone between Portland cement paste and aggregate had a significant influence on the flexural strength of SBR latex-modified mortars. The flexural strength of the modified mortar (with 15% SBR latex) was 8.6 MPa (66%) higher, compared with the mortar without SBR latex with a flexural strength of 5.2 MPa. Other authors [20] also recorded an increased flexural strength (by 18–30%), adhesion to the old concrete substrate (by 22–29%), as well as reduced open porosity, which causes a considerable change in capillary water absorption (by 18–56%) in SBR latex-modified calcium aluminate cement mortars.

An increase in the strength properties, as well as a decrease in the permeability or water absorption ratio, were observed in other cement-based materials, such as different types of concrete. Research results [19] have shown that with an increase in SBR latex content in concrete (from 5% to 20%), the flexural strength increased from 8.0 MPa to 11.5 MPa, and permeability (total charge passed) decreased from 1575 C to 847 C. It constitutes a growth in flexural strength by 20–50% and a drop in permeability by 5–49%, compared with concrete without SBR latex. Another study [21] reported a maximum increase of 21% in the compressive strength of concrete samples (with 15% SBR latex content), and a 36% increase in flexural strength after 28 days of hardening. Other authors recorded a maximum increase of 14% in compressive strength and a maximum decrease of 44% in water absorption of concrete samples (with 20% SBR latex content) after 28 days of hardening [22].

Studies [23] on SBR latex modified high strength class (C50/C60) concrete have shown that the minimum (19 MPa) and the maximum (66 MPa) compressive strength values were obtained in concrete with 1% and 8% SBR latex content, respectively. Concrete with 8% SBR latex content also demonstrated higher (by 25%) maximum values of splitting tensile strength (4.8 MPa), compared with concrete without SBR latex (3.6 MPa). The authors highlight a significant decrease (40–49%) in the water absorption rate of high-strength concrete modified with SBR latex and subsequently improved results of frost resistance after 30 cycles, with the decrease in scaling from 1.11 kg/m^2^ (for concrete without SBR latex) to 0.08 kg/m^2^ (for concrete with 8% SBR latex).

The newest research [24] on the properties of high-performance concrete has indicated that small amounts of SBR latex (0–7%) significantly enhanced flexural and tensile strengths, thermal resistance, and durability. Microstructural analysis (SEM, TGA, and XRD) confirmed a denser interfacial transition zone and reduced porosity in SBR latex-modified high-performance concrete samples.

The properties of different types of concrete modified with SBR latex were investigated in some other works. Authors reported decreased compressive [25] and flexural strengths due to increased porosity [26], better acid resistance [27] due to the presence of closed pores or reduced early-age compressive strength, but increased at the age of 28 days, as well as improved water sorption [28] in SBR latex modified ordinary concrete samples. In the studies [29,30,31] of concrete samples with lightweight aggregates or rubber waste aggregates, SBR latex appeared to be a positive surface modifier that ensures good wetting of hydrophobic surfaces.

Roller-compacted concrete is another suitable field where SBR latex can be successfully applied. Data from the early research [32] has shown that SBR latex-modified mixes achieved a good bond with the old concrete surface. The same authors [33] have concluded that the addition of SBR latex reduced the compressive and flexural strengths of roller-compacted concrete, mainly due to the air entrainment in the mix. They reported that the optimal amount of SBR latex to obtain high bond strength values was 10%.

Three-dimensional concrete is currently one of the most popular research areas in civil engineering. The results of fresh SBR latex-modified mixes obtained by the authors [34] confirmed excellent flowability, extrudability, workability, and open time, which are required for 3D printed materials. The SBR latex-modified mixes were suitable to serve as materials for 3D construction. Authors [35] reported that the incorporation of SBR latex into the 3D mixes was more efficient than the use of air-entraining agents to control the decrease in bond strength due to the freeze–thaw cycles. It was explained by the improved flexibility of the modified mortars, which accommodate deformations along the interfaces during freeze–thaw cycles.

The analysis of literature sources has shown that different properties of SBR latex-modified cement-based material samples reported were usually measured at 3, 7, 14 and 28 days of hardening. In this research, the authors decided to investigate a combined effect of high early-strength Portland cement, characterized by an increased hydration rate, and SBR latex was able to slow down this process for a prolonged hardening period–up to 90 days in modified mortar samples.

This study covers the results of the effect of different amounts of SBR latex (5%, 10%, 15% and 20%) on the properties of modified mortar samples with a constant water-to-cement ratio prepared with high early-strength Portland cement 42.5 R. The properties, such as dry bulk density, ultrasonic pulse velocity, capillary water absorption, compressive and flexural strengths, as well as toughness, after 3 different hardening periods (7, 28, and 90 days) of the mortar samples were investigated. The applied mathematical–statistical methods allowed a detailed prognosis of the dependence between the dry bulk density and the strength properties of modified mortar samples. The combination of 42.5 R strength class Portland cement with the SBR latex in amounts ranging from 5% to 20% seems to be suitable for designing durable structures with specific properties.

## 2. Materials and Methods

### 2.1. Materials

Local Portland cement CEM I 42.5 R, produced by the Lithuanian company Akmenes cementas and complying with standard LST EN 197-1:2011 [36] requirements, was used for the preparation of mortars. The mineral composition of cement clinker is presented in Table 1 (without gypsum), and the main properties are presented in Table 3.

Local natural washed sand of 0/4 fraction (sand particles 0–4 mm) from the Lithuanian company Zvyro karjerai quarry complying with the requirements of standard LST EN 12620:2003 [37] was used as an aggregate. The particle size distribution of sand is presented in Table 2, and the main properties are given in Table 3. The amounts of oxides in Portland cement and sand are presented in Table 4.

Tap water (temperature 20 °C) was used to prepare the mortars.

The new generation SBR latex 3642 (Laticrete, New Haven, CT, USA) was used for the modification of mortars. The main properties of SBR latex are presented in Table 5.

The water-to-cement ratio of all mortar mixtures was constant (0.125). It was ensured by taking into account the amounts of solid particles in SBR latex (see Table 5) and proportionally reducing the amount of water in the mortar mixture. The cement and sand ratio of 1:3 was also constant. The designation and compositions of different mortar mixtures are presented in Table 6.

The mortar mixtures of different compositions were mixed in the E095 mixer of 5 litres capacity in the following manner. Portland cement and sand were dry mixed for 2 min (the mixer’s shaft speed was 62 rpm). Then, the required amount of water or water-SBR latex mixture was added to the mortar mixture and wet mixed for 1 more minute (the mixer’s shaft speed was also 62 rpm). After that, the mixer’s shaft speed was increased to 140 rpm and the mixing procedure was continued for 3 more minutes. The prepared mortar mixtures were poured into the prism-shaped steel moulds (40 × 40 × 160 mm^3^) and compacted on a vibrating table for 20 s. The compacted mortar mixtures were cured in steel moulds, which were placed in the climatic chamber (relative humidity 65 ± 5%, temperature 20 ± 2 °C) for 2 days. After that, the hardened mortar samples were demoulded and kept in the same climatic chamber (relative humidity 65 ± 5%, temperature 20 ± 2 °C) for the required number of days until the measurements were taken.

### 2.2. Methods

The dry bulk density of hardened mortar samples was determined according to the requirements of the standard LST EN 1015-10:2002 [38]. Three prism-shaped samples of each mortar mixture were measured. The mortar samples were dried in a ventilated oven at the temperature of 75 ± 5 °C and weighed using the electronic balance EG 4200-2NM (KERN & SOHN GmbH, Balingen, Germany) with a weighing capacity of 4.2 kg and an accuracy of 0.01 g. The dry bulk density was calculated by the following equation:

(1) $$\rho =\frac{m}{V}$$

where *m* is the mass of the dry sample, kg; and *V* is the total volume of the sample, m^3^.

The structure development in hardened mortar samples (7, 28, and 90 days) was observed by applying the ultrasonic pulse velocity method (test instrument Pundit 7 (Proceq AG, Zurich, Switzerland), according to the requirements of the standard LST EN 12504-4:2003 [39]. Three prism-shaped samples of each mortar mixture were measured. The samples were placed between the ultrasonic transmitter and ultrasonic receiver (operating frequency 54 kHz) at opposite points. Vaseline was used to ensure good contact between the sample surfaces and the transducers. The ultrasonic pulse velocity was calculated by the equation [40]:

(2) $$V=\frac{l}{t}\cdot {l0}^{6}$$

where *l* is the distance between cylindrical heads, m; and *t* is the time of pulse spread, s.

The capillary water absorption coefficient of hardened mortar samples was determined after 7, 28, and 90 days according to the requirements of the standard LST EN 1015-18:2003 [41]. Three prism-shaped samples of each mortar mixture were measured. They were cured for 2 days in moulds and 5 days after demoulding at 20 ± 2 °C (relative humidity 95 ± 5%) and then for 21 days at 20 ± 2 °C (relative humidity 65 ± 5%). The samples were coated with a specified sealing material and broken into two halves. These halves were immersed with the broken surface down into distilled water at a depth of 10 mm. The samples were weighed after 10 and 90 min of soaking. The capillary water absorption coefficient was calculated by the following equation:

C = 0.1 (*M*2 − *M*1), (3)

where *M*1 is the mass of the mortar specimen after 10 min immersion in water, kg; and *M*2 is the mass of the mortar specimen after 90 min immersion in water, kg.

The flexural and compressive strengths of hardened mortar samples were determined at 7, 28, and 90 days according to the requirements of the standard LST EN 1015-11:2019 [42]. An electromechanical testing machine H200kU (Tinius Olsen Ltd, Salfords, England) with a load capacity of 200 kN and a load measurement error of ±0.5% was used.

**Figure 1:**
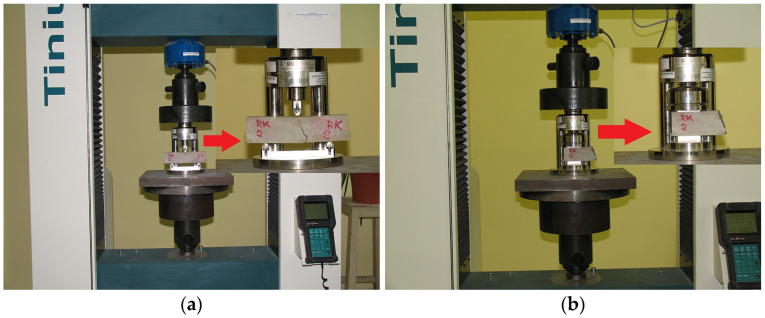
Testing of hardened mortar samples: (**a**)—flexural strength; (**b**)—compressive strength.

The flexural strength of 3 prism-shaped samples of each mortar mixture was tested. The 3-point bending method was used in the test (Figure 1a). The applied load was 40 N/s. The flexural strength was calculated by the following equation:

(4) $$f_{f}=1.5\;\frac{F\cdot l}{b\cdot d²}$$

where *F* is the maximum flexural force, N; *l* is the distance between the supports, mm; *b* is the width of the mortar sample, mm; and *d* is the thickness of the mortar sample, mm.

The compressive strength of 6 samples of each mortar mixture was tested using the prism halves remaining after the flexural strength testing (Figure 1b). The applied load was 400 N/s. The compressive strength was calculated by the equation:

(5) $$fc=\frac{F}{A}$$

where *F* is the maximum compressive force, N; and *A* is the cross-sectional area of the sample, mm^2^.

The toughness of hardened mortar samples was calculated at 7, 28 and 90 days using the results of flexural and compressive strengths of the mortar samples from the equation [43]:

(6) $$k=\frac{f_{c}}{f_{f}}$$

where *f_c_* is the compressive strength, MPa; and *f_f_* is the flexural strength, MPa.

### 2.3. Statistical Evaluation of Research Results

Mathematical–statistical methods and the software STATISTICA 6 were used to process the obtained experimental data.

The dispersion characteristics used for the evaluation of the normal distribution of the properties of hardened mortar samples were the arithmetic mean (*x*) and the standard square deviation (*S_x_*), which were defined by means of the following statistical formulas:

(7) $$\bar{x}=\frac{1}{n}\sum^{n}x_{i}$$

(8) $$S_{x}=\sqrt{\frac{\sum_{i=1}^{n}(x_{i}-\bar{x}{)}^{2}}{n-1}}$$

where *n* is the number of tests in the sample; and *x*_1_, *x*_2_,…, *x_n_* are the results of observations.

To evaluate the reliability of the dispersion of experimental data with a given probability, it is necessary to estimate the confidence intervals (*A*) of the mean of the obtained values:

(9) $$\overset{-}{x}-\frac{t_{\alpha }\cdot S_{x}}{\sqrt{n}}<A<\overset{-}{x}+\frac{t_{\alpha }\cdot S_{x}}{\sqrt{n}}$$

where *t_α_* is Student’s criteria.

## 3. Results

### 3.1. Dry Bulk Density

**Figure 2:**
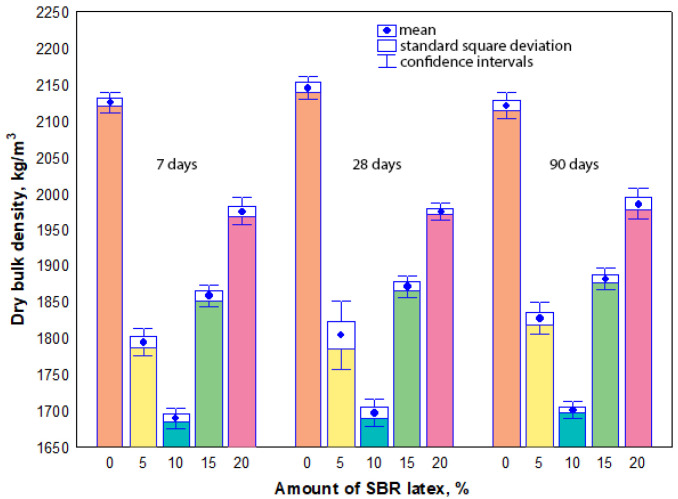
Relationship between the dry bulk density values and the amount of SBR latex.

The measurement results have shown that dry bulk density values of all mortar samples were influenced considerably by the amount of SBR latex, not by the sample curing time. The arithmetic mean of dry bulk density values ranged from 2125 kg/m^3^ to 2071 kg/m^3^ in the mortar samples without SBR latex at the curing time of 7 to 90 days, respectively. When SBR latex was added to the mortar samples from 5% to 10%, the arithmetic mean of dry bulk density values decreased and ranged from 1795 kg/m^3^ to 1828 kg/m^3^, and from 1690 kg/m^3^ to 1702 kg/m^3^, respectively, depending on the curing time. A different tendency in the results was observed when the amount of SBR latex in the mortar samples was increased from 15% to 20%. The arithmetic mean of dry bulk density values slightly increased and ranged from 1860 kg/m^3^ to 1882 kg/m^3^, and from 1971 kg/m^3^ to 1986 kg/m^3^, at 7 and 90 days, respectively. The maximum decrease in the arithmetic mean of dry bulk density values was observed in the mortar samples containing 10% SBR latex. Depending on the curing time of these samples, it was 21–22% lower than the arithmetic mean of dry bulk density values of the mortar samples without SBR latex. The dry bulk density results are shown in Figure 2.

### 3.2. Ultrasonic Pulse Velocity

**Figure 3:**
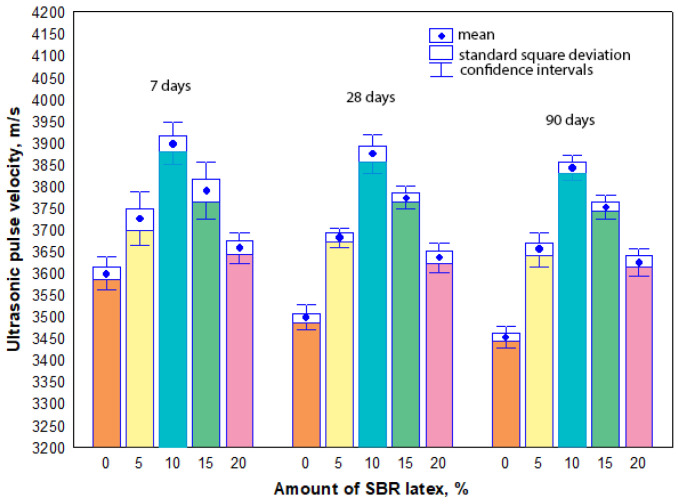
Relationship between the ultrasonic pulse velocity values and the amount of SBR latex.

The measurement results have shown that ultrasonic pulse velocity values of all mortar samples were influenced more significantly by the amount of SBR latex in the samples, not by the curing time. The arithmetic mean of ultrasonic pulse velocity values ranged from 3600 m/s to 3455 m/s in the mortar samples without SBR latex at 7 and 90 days, respectively. When SBR latex was added to the mortar samples from 5% to 10%, the arithmetic mean of ultrasonic pulse velocity values slightly increased and ranged from 3725 m/s to 3655 m/s and from 3898 m/s to 3842 m/s, respectively, depending on the curing time. A different tendency in the results was observed when the amount of SBR latex in the mortar samples was increased from 15% to 20%. The arithmetic mean of ultrasonic pulse velocity values slightly decreased and ranged from 3898 m/s to 3842 m/s and from 3659 m/s to 3617 m/s, respectively, depending on the curing time. The maximum increase in the arithmetic mean of ultrasonic pulse velocity values was observed in the mortar samples containing 10% SBR latex. Depending on the curing time of these samples, it was 8–10% higher than the arithmetic mean of ultrasonic pulse velocity values of the mortar samples without SBR latex. The results of ultrasonic pulse velocity tests are shown in Figure 3.

### 3.3. Capillary Water Absorption

**Figure 4:**
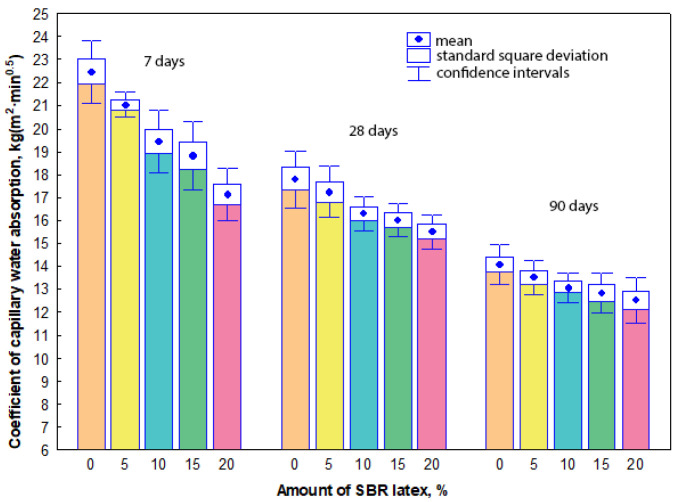
Relationship between the capillary water absorption values and the amount of SBR latex.

The measurement results have shown that capillary water absorption values of all mortar samples were influenced more significantly by the curing time of the samples, not by the amount of SBR latex. The arithmetic mean of the coefficient of capillary water absorption values ranged from 22.5 kg(m^2^·min^0.5^) to 14.1 kg(m^2^·min^0.5^) in the mortar samples without SBR latex at the curing time from 7 to 90 days, respectively. When SBR latex was added to the mortar samples from 5% to 10%, the arithmetic mean of the coefficient of capillary water absorption values slightly decreased and ranged from 20.9 kg(m^2^·min^0.5^) to 13.5 kg(m^2^·min^0.5^) and from 19.4 kg(m^2^·min^0.5^) to 13.1 kg(m^2^·min^0.5^), respectively, depending on the curing time. The same tendency of the results was observed when the amount of SBR latex in the mortar samples was increased from 15% to 20%. The arithmetic mean of the coefficient of capillary water absorption values decreased and ranged from 18.8 kg(m^2^·min^0.5^) to 12.8 kg(m^2^·min^0.5^) and from 17.1 kg(m^2^·min^0.5^) to 12.5 kg(m^2^·min^0.5^), respectively, depending on the curing time. The maximum decrease in the arithmetic mean of the coefficient of capillary water absorption values was observed in the mortar samples containing 20% SBR latex. Depending on the curing time of these samples, it was 24–11% lower than the arithmetic mean of the coefficient of capillary water absorption values of mortar samples without SBR latex. The coefficients of capillary water absorption results are shown in Figure 4.

### 3.4. Flexural Strength

**Figure 5:**
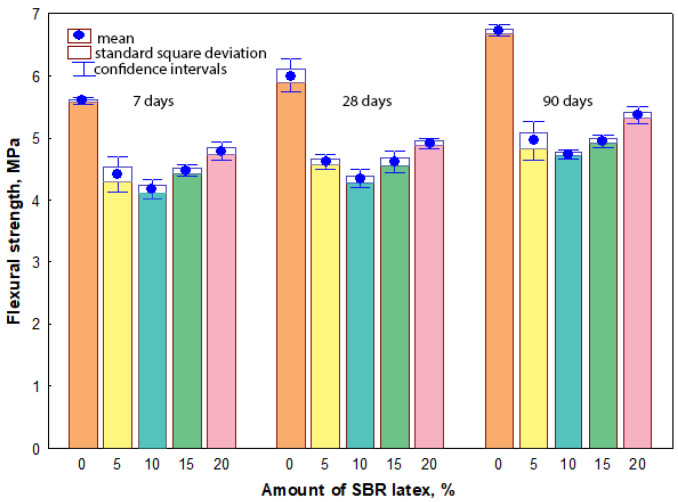
Relationship between the flexural strength values and the amount of SBR latex.

**Figure 6:**
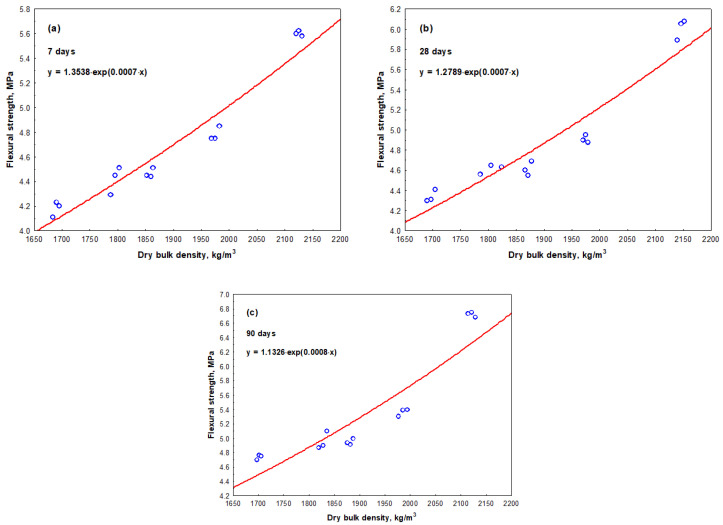
Relationship between the flexural strength and dry bulk density of the mortar samples: (**a**)—after 7 days; (**b**)—after 28 days; (**c**)—after 90 days.

The measurement results have shown that flexural strength values of all mortar samples were influenced both by the amount of SBR latex and the curing time. The arithmetic mean of flexural strength values ranged from 5.6 MPa to 6.7 MPa in the mortar samples without SBR latex, when the curing time was from 7 to 90 days, respectively. When SBR latex was added to the mortar samples from 5% to 10%, the arithmetic mean of flexural strength values decreased and ranged from 4.4 MPa to 5.0 MPa and from 4.2 MPa to 4.8 MPa, respectively, depending on the curing time. The same tendency of the results was observed when the amount of SBR latex in the mortar samples was increased from 15% to 20%. The arithmetic mean of flexural strength values slightly increased and ranged from 4.5 MPa to 5.0 MPa and from 4.8 MPa to 5.4 MPa, respectively, depending on the curing time. The maximum decrease in the arithmetic mean of flexural strength values was observed in the mortar samples containing 10% SBR latex. Depending on the curing time of these samples, it was 25–29% lower than the arithmetic mean of flexural strength values of the mortar samples without SBR latex. The flexural strength results are shown in Figure 5. The relationship between the flexural strength and dry bulk density of the mortar samples is shown in Figure 6.

The dependence of flexural strength values at 7, 28 and 90 days is described by the exponential Equations (10), (11) and (12), respectively:

*f_f_*_7_ = 1.3538·exp (0.0007·*ρ*_7_), (10)

with a determination coefficient *r*^2^ = 0.858 and standard deviation *S_r_* = 0.42362 MPa, which have shown that the variation in flexural strength value at an average of 86% depends on the dry bulk density value of the mortar sample; and

*f_f_*_28_ = 1.2789·exp (0.0007·*ρ*_28_), (11)

with a determination coefficient *r*^2^ = 0.835 and standard deviation *S_r_* = 0.42150 MPa, which have shown that the variation in flexural strength value at an average of 84% depends on the dry bulk density value of the mortar sample; as well as

*f_f_*_90_ = 1.1326·exp (0.0008·*ρ*_90_), (12)

with a determination coefficient *r*^2^ = 0.7483 and standard deviation *S_r_* = 0.44580 MPa, which have shown that the variation in flexural strength value at an average of 75% depends on the dry bulk density value of the mortar sample.

### 3.5. Compressive Strength

**Figure 7:**
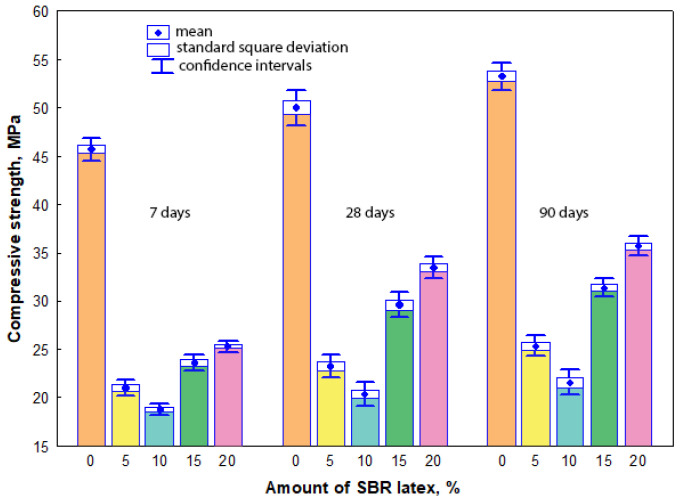
Relationship between the compressive strength values and the amount of SBR latex.

**Figure 8:**
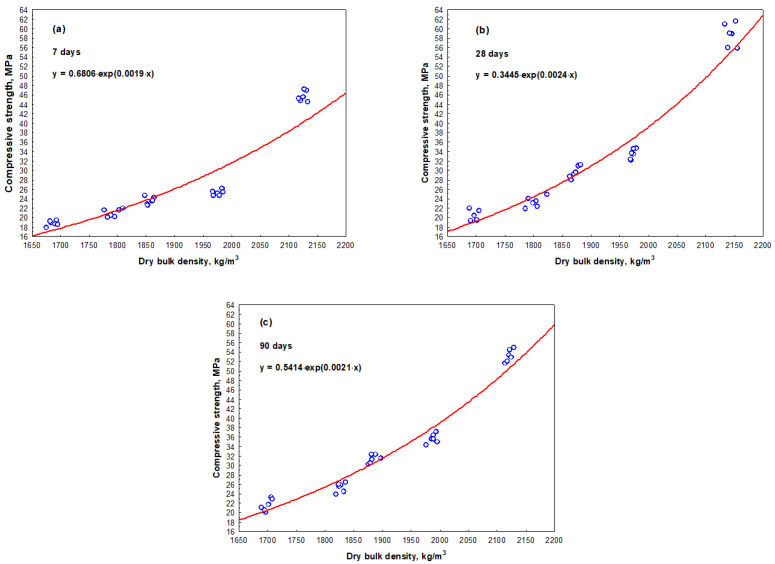
Relationship between the compressive strength and the dry bulk density of mortar samples: (**a**)—after 7 days; (**b**)—after 28 days; (**c**)—after 90 days.

The measurement results have shown that the compressive strength values of all mortar samples were influenced both by the amount of SBR latex and the curing time. The arithmetic mean of the compressive strength values ranged from 45.5 MPa to 53.3 MPa in the mortar samples without SBR latex, when the curing time was from 7 to 90 days, respectively. When SBR latex was added to the mortar samples from 5% to 10%, the arithmetic mean of compressive strength values decreased considerably and ranged from 20.2 MPa to 25.8 MPa and from 18.6 MPa to 22.6 MPa, respectively, depending on the curing time. A different tendency in the results was observed when the amount of SBR latex in the mortar samples was increased from 15% to 20%. The arithmetic mean of compressive strength values slightly increased and ranged from 23.5 MPa to 31.4 MPa and from 25.2 MPa to 35.6 MPa, respectively, depending on the curing time. The maximum decrease in the arithmetic mean of the compressive strength values was recorded in the mortar samples containing 10% SBR latex. Depending on the curing time of these samples, it was 48–58% lower than the arithmetic mean of compressive strength values of the mortar samples without SBR latex. The results of compressive strength are shown in Figure 7. The relationship between the compressive strength and dry bulk density of the mortar samples is shown in Figure 8.

The dependence of the compressive strength values (after 7, 28 and 90 days of curing) is described by the exponential Equations (13), (14) and (15), respectively:

*f_c_*_7_ = 1.3538·exp (0.0007·*ρ*_7_), (13)

with a determination coefficient *r*^2^ = 0.923 and standard deviation *S_r_* = 0.18338 MPa, which have shown that the variation in compressive strength value at an average of 92% depends on the dry bulk density value of the mortar sample; and

*f_c_*_28_ = 1.2789·exp (0.0007·*ρ*_28_), (14)

with a determination coefficient *r*^2^ = 0.968 and standard deviation *S_r_* = 0.14579 MPa, which have shown that the variation in compressive strength value at an average of 97% depends on the dry bulk density value of the mortar sample; as well as

*f_c_*_90_ = 1.1326·exp (0.0008·*ρ*_90_), (15)

with a determination coefficient *r*^2^ = 0.957 and standard deviation *S_r_* = 0.20508 MPa, which have shown that the variation in compressive strength value at an average of 96% depends on the dry bulk density value of the mortar sample.

### 3.6. Toughness

**Figure 9:**
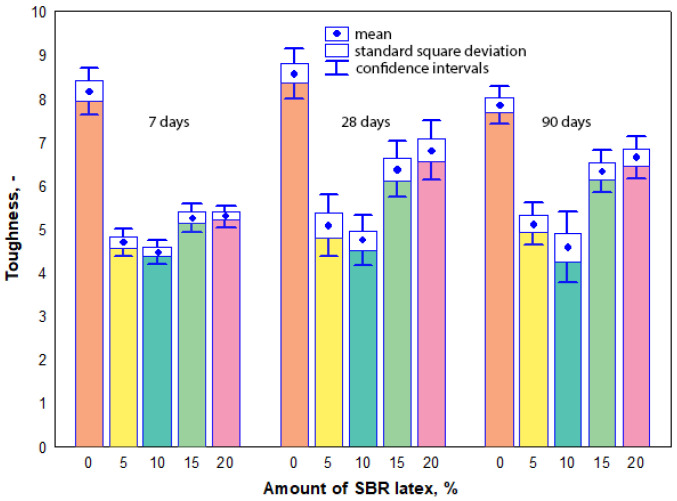
Relationship between the toughness values and the amount of SBR latex.

Toughness is the ratio of compressive to flexural strengths of the modified mortar samples with lower values indicating better toughness. The measurement results have shown that the toughness values of all mortar samples were influenced both by the amount of SBR latex and the curing time of the samples. The arithmetic mean of toughness values ranged from 8.6 to 7.9 in the mortar samples without SBR latex, when the curing time was from 7 to 90 days, respectively. When SBR latex was added to the mortar samples from 5% to 10%, the arithmetic mean of toughness values decreased considerably and ranged from 4.7 to 5.1 and from 4.5 to 4.8, respectively, depending on the curing time. A different tendency of the results was observed when the amount of SBR latex in the mortar samples was increased from 15% to 20%. The arithmetic mean of toughness values slightly increased and ranged from 5.3 to 6.4 and from 5.3 to 6.8, respectively, depending on the curing time. The maximum decrease in the arithmetic mean of toughness values was observed in the mortar samples containing 10% SBR latex. Depending on the curing time of these samples, it was 45–42% lower than the arithmetic mean of toughness values of the mortar samples without SBR latex. The toughness results are shown in Figure 9.

## 4. Discussion

Air can be easily entrained during the mixing of mortars modified with SBR latexes due to the presence of hydrophobic groups, thus causing the formation of air bubbles in the macrostructure of the mortar [18].

It was observed in our research that the presence of air bubbles caused a considerable decrease in the dry bulk density of the modified mortar samples, especially when the amount of SBR latex was up to 10% (Figure 2). This decrease in the dry bulk density of the mortar samples (more air voids) naturally causes the increase in ultrasonic pulse velocity (Figure 3) or decrease in strength properties (Figure 5 and Figure 7) or toughness (Figure 9).

The results obtained in our study differed from the results reported by other authors [44], who found that the bulk density increased by 15% with an increase in the amount of SBR latex from 0% to 10%. The increase in the bulk density influenced the growth of compressive strength values of SBR-modified mortars almost three times (the amount of SBR latex was from 0% to 15%). The authors stated that SBR latex particles interact with cement paste, resulting in superior bonding between the cement paste and the aggregates. The rapid decrease in the bulk density and the compressive strength values was recorded only when the amount of SBR latex was above 10% and 15%. In our experiments, the dry bulk density values of SBR latex-modified mortar samples decreased considerably from 2130 kg/m^3^–2070 kg/m^3^ to 1800 kg/m^3^–1830 kg/m^3^ or 1690 kg/m^3^–1700 kg/m^3^ at even lower amounts of SBR latex, 5–10%, respectively. These findings correspond to the research results obtained by other authors [16,20,39]. In our tests, the curing time practically did not influence the dry bulk density values of SBR latex-modified mortar samples.

The authors in [45] also observed tendencies similar to our results. The compressive and flexural strengths of modified mortar samples containing 7–10% SBR latex were directly proportional to the apparent bulk density. The growth in the apparent bulk density values of modified mortar samples was observed when the amount of SBR latex was from 10% to 20%. The properties of SBR latex-modified mortar samples were influenced by the film thickness of SBR latex, Portland cement hydrates, and the formed structure between the organic and inorganic phases.

In our tests, the dependence of the compressive strength values after 7, 28 and 90 curing days was described by exponential equations, which have shown that the variation in compressive strength value at an average of 92%, 97%, and 96% depends on the dry bulk density value of the mortar sample. The compressive strength values of all mortar samples were influenced both by the amount of SBR latex and the curing time. The compressive strength values of the mortar samples without SBR latex measured at 7 days, 28 days, and 90 days were 45.8 MPa, 50.9 MPa, and 53.3 MPa, respectively. The compressive strength values decreased to 21.2 MPa, 23.5 MPa and 25.4 MPa in the mortar samples with 5% SBR latex at 7 days, 28 days and 90 days, respectively. The decrease in strength properties could be explained by the constant water-to-cement ratio, which led to higher porosity in modified mortar samples when the amount of SBR latex increased [46]. At 7 days, 28 days, and 90 days, the lowest compressive strength values of 18.5 MPa, 20.4 MPa, and 22.2 MPa, respectively, were obtained in the mortar samples modified with 10% SBR latex. This tendency was also observed by other authors [19]. At 28 days, the compressive strength of SBR latex (15%) modified mortar samples decreased by 47% (from 47 MPa to 28 MPa), compared to the average compressive strength of the mortar samples without SBR latex. This decrease was influenced by the changes in the interfacial transition zone between aggregate particles and Portland cement paste.

In our tests, different results were obtained when the amount of SBR latex in the mortar samples was increased to 15% or 20%. Depending on the curing time, the compressive strength values slightly increased and ranged from 23.5 MPa to 31.4 MPa and from 25.2 MPa to 35.6 MPa, respectively. Other authors [43] also observed decreased compressive strength values (from 39 MPa–61 MPa to 23 MPa–40 MPa or 15 MPa–28 MPa) in modified mortar samples after 3, 7, and 28 curing days with an increase in SBR latex content from 0% to 5% or 10%, respectively. Different results were obtained in the study [47], reporting that compressive strength values of modified mortar samples decreased by about 31% when the amount of SBR latex was 20%.

In our research, the dependence of flexural strength values after the same curing time was also described by exponential equations, which have shown that the variation in flexural strength value at an average of 86%, 84%, and 75% depends on the dry bulk density value of the mortar sample. The flexural strength values of all mortar samples were also influenced both by the amount of SBR latex and the curing time of the samples. At 7 days, 28 days, and 90 days, the flexural strength values of the mortar samples without SBR latex were 5.6 MPa, 6.0 MPa, and 6.8 MPa, respectively. The authors in [43] also stated that longer curing times of mortar samples caused higher flexural strength values and the toughness of modified mortars improved with an increase in the amount of SBR latex.

At 7 days, 28 days, and 90 days, the flexural strength values decreased to 4.4 MPa, 4.6 MPa, and 5.0 MPa, respectively, in the mortar samples containing 5% SBR latex. At 7 days, 28 days, and 90 days, the lowest flexural strength values of 4.2 MPa, 4.3 MPa, and 4.7 MPa, respectively, were obtained in the mortar samples containing SBR 10% latex. The authors in [43] also reported decreased flexural strength values (from 6.9 MPa–8.5 MPa to 5.5 MPa–7.3 MPa or 4.0 MPa–5.5 MPa) of modified mortar samples after 3, 7, and 28 curing days, with an increase in the amount of SBR latex from 0% to 5% or 10%, respectively. Another study [47] reported that when the amount of SBR latex in modified mortar samples was 8%, the flexural strength values decreased by 29%. The SBR latex film occupied the site of hydration products resulting in lower flexural strength values of modified mortar samples.

In our tests, different flexural strength results were also obtained when the amount of SBR latex in the mortar samples increased to 15% or 20%. At 7, 28, and 90 curing days the flexural strength values of modified mortar samples slightly increased and ranged from 4.5 MPa, 4.6 MPa, and 5.0 MPa to 4.8 MPa, 4.9 MPa, and 5.4 MPa, respectively. These results could have been caused by the SBR latex film covering Portland cement crystals and filling in the pores, thus increasing the flexural strength of the modified mortar samples [47].

Our measurements have shown that the toughness values of all mortar samples were influenced more by the amount of SBR latex and less by the curing time of the mortar samples. At 7 days, 28 days and 90 days, the toughness values in the mortar samples without SBR latex were 8.2, 8.6, and 7.9, respectively. At 7 days, 28 days, and 90 days, the toughness values in the modified mortar samples containing 5% SBR latex decreased to 4.7, 5.1, and 5.1, respectively. The decrease in toughness could also be explained by the constant water-to-cement ratio, which caused higher porosity in modified mortar samples as the amount of SBR latex increased [46].

At 7 days, 28 days, and 90 days, the lowest toughness values of 4.5, 4.8, and 4.6, respectively, were obtained in the modified mortar samples containing 10% SBR latex. This is the result of the formation of coherent polymer films, which prevent the development of microcracks [43].

Our tests produced different results when the amount of SBR latex in the mortar samples was increased to 15% or 20%. The toughness values slightly increased and ranged from 5.3 to 6.4 and from 5.3 to 6.8, depending on the curing time. The authors in [43] also stated that longer curing times of mortar samples generated higher flexural strength values and the toughness of modified mortars improved with a higher SBR latex content.

In all cases, the nonlinear relation between SBR latex content and the properties of modified mortar samples can be explained by the changed surface tension in fresh mortar mixtures. The reduced surface tension, influenced by hydrophobic groups of SBR latex, allows the formation of air bubbles during the mixing process, which can affect the macrostructure of modified mortar samples. The concentration of hydrophobic groups at the surface of fresh mortar mixtures can quickly reach full saturation (especially at higher amounts of SBR latex); therefore, further reduction in the surface tension in fresh mortar samples is not possible [18].

The durability of the mortars depends on the microstructure and could be closely related to water absorption or capillary water absorption. In the study [20], the capillary water absorption of mortar samples containing from 3% to 9% SBR latex was measured. These amounts of SBR latex considerably reduced (from 18% to 56%) the capillary water absorption values of modified mortar samples after 7 days of curing. The authors explained this decrease by the reduced open porosity of the modified mortar samples, as the pores were filled with SBR latex, which also served as bridges for microcracks developed in the cement paste during the hydration process. In our experiments, it was observed that the coefficient of capillary water absorption decreased not only with an increase in SBR latex content but also with longer curing time. In the research, only this measurement showed a linear relation between the amount of SBR latex and the measured property of modified mortar samples. After 90 days, the mortar samples modified with 20% SBR latex showed the lowest values (24% lower compared to the samples without SBR latex). The authors in [44] also declared a decrease in water absorption with the increase in SBR latex content in the mortar samples. The samples containing 10% to 20% SBR latex had water absorption values ranging from 2.7% to 1.3%.

## 5. Conclusions

This study analyzed the effect of SBR latex on the properties of mortars (constant water-to-cement ratio) prepared with 42.5 R Portland cement at different curing times (7, 28, and 90 days). This experimental study led to the following conclusions:

- The maximum decrease in the arithmetic mean of dry bulk density values was found in the mortar samples containing 10% SBR latex. It was 21–22% lower than the arithmetic mean of dry bulk density values of the mortar samples without SBR latex, depending on the curing time;
- The maximum increase in the arithmetic mean of ultrasonic pulse velocity values was found in the mortar samples containing 10% SBR latex. Depending on the curing time of these samples, it was 8–10% higher than the arithmetic mean of ultrasonic pulse velocity values of the mortar samples without SBR latex;
- The maximum decrease in the arithmetic mean of the coefficient of capillary water absorption values was found in the mortar samples containing 20% SBR latex. Depending on the curing time of these samples, it was 24–11% lower than the arithmetic mean of the coefficient of capillary water absorption of mortar samples without SBR latex;
- The maximum decrease in the arithmetic mean of the flexural strength values was found in the mortar samples containing 10% SBR latex. Depending on the curing time of these samples, it was 25–29% lower than the arithmetic mean of flexural strength values of the mortar samples without SBR latex. Statistical analysis has shown that after 7, 28, and 90 days of hardening, the variation in the flexural strength values of modified mortar samples at an average of 86%, 84%, and 75% depends on dry bulk density values;
- The maximum decrease in arithmetic mean of the compressive strength values was fixed in the mortar samples with the amount of SBR latex of 10%. Depending on the curing time of these samples, it was 48–58% lower than the arithmetic mean of compressive strength values of the mortar samples without SBR latex. Statistical analysis has shown that the variation in the compressive strength values of modified mortar samples after 7, 28, and 90 days of hardening, at an average of 92%, 97%, and 96%, depends on the dry bulk density values;
- The maximum decrease in the arithmetic mean of toughness values was found in the mortar samples containing 10% SBR latex. Depending on the curing time of these samples, it was 46–37% lower than the arithmetic mean of toughness values of the mortar samples without SBR latex;
- The obtained research results can be useful in designing structures for outdoor or aggressive environments—swimming pools, industrial floors, or paving blocks.

In the near future, the authors are planning to focus more on the durability research of SBR latex-modified mortar samples, as the results of porosity and kinetics of full water absorption are showing positive directions.

## Figures and Tables

**Table 1:** Mineral composition of cement clinker.

Mineral	Amount (wt. %)
tricalcium silicate	57.80
β-dicalcium silicate	22.15
tricalcium aluminate	6.65
tetracalcium aluminoferrite	13.40

**Table 2:** Particle size distribution of sand (fraction 0/4).

Sieve Aperture, mm	Passed, %
5.600	100.0
4.000	99.20
2.000	91.67
1.000	75.73
0.500	46.47
0.250	10.13
0.125	2.37
<0.063	0.60

**Table 3:** Physical properties of Portland cement and sand.

Physical Properties	Portland Cement	Sand
specific density (kg‧m^−3^)	3150	2650
Blaine fineness (m^2^‧kg^−1^)	380	−
median particle size (µm)	17.6	−
bulk density (kg‧m^−3^)	−	1670
water absorption (wt. %)	−	≤1.0

**Table 4:** Chemical compositions of Portland cement and sand.

Material	Oxide Content (wt. %)								
	CaO	SiO_2_	Al_2_O_3_	Fe_2_O_3_	MgO	SO_3_	K_2_O	Na_2_O	LOI
Portland cement	61.4	19.5	5.00	3.10	3.90	2.50	1.10	0.10	3.40
sand	2.05	90.4	4.02	0.65	0.49	-	1.00	0.34	1.02

**Table 5:** The main physical and chemical properties of SBR latex.

State	Colour	Smell	Boiling Temperature	Density	Miscibility with Water	Amount of Hard Particles
liquid	data	weak	100 °C (1.000 hPa)	1.0 g/cm^3^	mixable	about 50%

**Table 6:** Amounts of raw materials in mortar mixtures.

Raw Materials	Designation of Mortar Mixtures				
	CR0	CR5	CR10	CR15	CR20
Portland cement (kg)	0.900	0.900	0.900	0.900	0.900
sand (kg)	2.700	2.700	2.700	2.700	2.700
water (kg)	0.4500	0.4275	0.4050	0.3825	0.3600
SBR latex (vol.%/kg)	0/-	5/0.045	10/0.090	15/0.135	20/0.180

## Data Availability

The original contributions presented in the study are included in the article. Further inquiries can be directed to the corresponding author.
